# A Gossip with Women

**Published:** 1876-08

**Authors:** 


(*From Treasure Trove.*)

### *A GOSSIP WITH WOMEN.*

BY M. H. F.

---

### PETS, POKES AND PICTURES, FASHIONS AND FEMALES.

Year by year the keeping of "Pets" increases in New York. Throughout Europe it has always been a sort of mania, but not until lately has it been so much of a fashion with American women.

Now magnificent stores are superseding the dingy basements where some gloomy looking son of a maccaroni sold a few birds and dogs.

Everybody keeps canaries, almost everybody keeps a pet dog. To these are now added a parrot and oftentimes a monkey.

One very rarely sees the old fashioned pet, the French poodle. That weak eyed little wretch required so much time and labor t prevent his looking like a door mat, that he has been replaced in female affection by the "black and tan" or the "Scotch terrier" and now the "pug."

And of all the snub-nosed ruffians on four legs, commend me to an English Pug.

### MRS HOEY'S PUG.

Mrs. John Hoey goes about lugging one, the size of a small bull dog, a brindle yellow in color, a bandy legged, pompous unsatisfied canine whose nose has been mashed back behind its ears.

It must weigh 10 pounds, but carefully borne in her arms the lady takes it abroad for an airing.

Mrs. Hicks the famous equestrienne and belle of 14th street has some most rare and beautiful specimens of the Scotch terrier.

### CLAUDE AND HIS TOOTHACHE.

Mrs. Helmbold has a tiny, black and tan, who some time ago sat down on his tail and made the day a horror and the night a nuisance with his dismal wails.

When he first began his diabolical concerts the listeners took to tables and shelves and other high places to be out of the reach of hydrophobia.

But as he showed no disposition to bite, they began to study his complaint and finally found it was the toothache.

He would bury his head in the sofa-cushion and try to throw away his hind legs. When presented with the wish-bone of a chicken, he took one bite and retired to a position in the corner where he howled for an hour.

This could not be endured for long; so as doggie was a valuable specimen, a council was held, a sort of advisory council, and the result was Mr. Claude went off to have his teeth repaired, after the ravages of candy and sweet cake had done their sad work.

Poor Claude, he wished he had been born a bone hunter, to have lived on oyster shells and such other friends to canine dental arrangements. When snugly packed in an enveloping shawl, tied like a bundle with a string, a dreadful stick fastened into his jaws, he was made acquainted with the delights of tooth pulling and filling.

The result was satisfactory however. Claude came home a reconstructed animal, took to his candy with renewed vigor and appetite, and is probably by this time again a candidate for the dentists chair.

### PEDRO, THE TURTLE.

One of the oddest pets that ever existed belongs to a little girl in 71st street. A visitor met her at the gate last summer with what she supposed was a lunch box under her arm. To the lady's surprise a snake-like head ran out a little way from the lunch box, and the pet turned out to be a common mud turtle.

Some one had brought the creature home, a year or so before, and let it loose in the garden.

When the children began to play there, out from its hole came the turtle and singling out this child gave evidence of intelligence beyond belief.

The turtle was called Pedro, and the little girl had but to call that name two or three times at the back door, to see the old box waddling up from the bottom of the garden—directly to her. Any one else approaching or attempting familiarities, the clumsy feet were drawn promptly in, and the wise old head retired within its shell and no sign of life would Pedro give.

But let the child take it, the claws closed round one of her fingers, and the head, run out to its fullest extent of neck, would be waggled against her with every token of love a turtle could give.

There came a time during the summer when the little mistress was sick. The mother sitting in the dusk thought she saw a dark object moving in the room, and shortly after hearing a bumping on the stairs, went out and found it was Pedro returning to his hole in the yard.

Each day for weeks, with the opening of the outside door, in would walk Pedro. The girl generally found him waiting on the steps.

He would go to the stairs, stand upright in a corner and setting his little feet into the stair above pull himself up. This operation he would repeat till he reached the landing. If the door into the sick child's room was closed, he waited patiently to be allowed to enter.

When taken up and laid beside its mistress, it made no effort to move, but lay for hours with its solemn head wagging from side to side.

At night it waddled off to the stairs, and then the racket commenced, as it fell off each step in clumsy fashion, sounding like that famous clock in the Ingoldsby legends that went hopping after Mr. Ap Jones.

### DOMESTIC ENGLISH GIRLS.

It is a good sign in women to find them attached to pets.

In England, where the most lovable domestic women in the world exist, there seems to be no end of pets. Angora cats, all kinds of dogs, varieties of birds, and above all poll parrots, abound in all directions.

And what a difference there is between a family of grown up and growing girls in England and America.

The English girl is never seen walking up and down Oxford street or Regent street, as the American girl is met promenading Broadway or the Avenue.

The English girl puts on in the fall a simple jacket of cloth, a round walking hat, a stuff dress and a pair of strong boots, and in this costume she takes her constitutional. Straight out some unfrequented streets for miles and back again. And this she repeats daily, in the same trim, till warm weather bids her change her dress.

Mademoiselle the American would be horrified at wearing the same old things day in and week out.

Variety is the necessity of a girl's wardrobe. Would the New York young lady think of walking off up the Boulevard St. Nicholas, any unbuilt up street. Not she! Broadway or the Fifth Avenue is he locality to air all the frills and fixings of our young women.

On her French heels it is a very ticklish matter to toddle up and down a dozen blocks, and she would never put on such abominations as broad-soled, low-heeled, wide English walking boots.

The English girls at home were new revelations to me. I had been used to the eternally bickering, hair crimping, fellow discussing New York girl. And it was something novel to see a group of highly educated girls gathered in a sewing room, busy in a dozen different ways while one of them read the then new novel of "Middlemarch."

Another day in an entirely different grade of society, among girls of moderate education and limited means, exactly the same scene was reproduced. In weeks spent among these families, I never heard the unfailing topic of American conversation introduced—Gentlemen under consideration as lovers—till finally I asked about it, and one girl colored and said, "Agnes is engaged and I suppose I shall be next Spring."

And another laughed and answered:

"Oh there's a country Squire comes courting us. We don't yet know which one of us. As there are seven eligible parties, its a little uncertain and makes it all the more interesting."

These girls were not slying off to such post offices as "Godfrey's" on Broadway getting notes from Tom, Dick and Harry. They would have been horrified at the enormity of flirting into an acquaintance with a strange man on the street. I'd like to know how many New York girls feel so.

### THE RELIGION OF DRESS.

The labor and discontent of the women of America to out-dress each other is the curse of the sisterhood.

When a lady determines to be conspicuous for the richness and elegance of her dress, she assumes the labors of Hercules and her reward is discontent. She can never get herself up so magnificently but some one will be a little more gorgeous.

"Oh vanity and vexation of spirit!" Think of those happywomen who wear to-day the high caps and bodices, the striped petticoats and clocked stockings, precisely the same as their mothers, grandmothers, and great grandmothers wore 'em five hundred years ago in the Cantons of Switzerland. I wish I was in a Canton in Switzerland and emancipated from this slavery of dress.

After this protest against the bother of clothes it will be perfectly in character to go directly into inky spasms over the last new thing.

There are not many to describe. The rough walking suits still predominate.

There's a roughness and a delicacy just now blended in equal proportions.

If I were going to get a lady put up properly at a drug store or some such place I should say two parts crash toweling, sand paper and old iron, and one part glacie silk, blush roses and glycerine.

They get the lumpiest cloth to be found, they cord it with little less than velvet covered cable. They use clasps of metal that would couple freight cars, and then they top off this costume with flying scarfs of illusion and delicate flowers and cream colored velvets, and the extract of wood violet.

Feathers are superseding flowers for street wear, but still word comes that the Spring hat will be not only large, but a regular flower show.

A friend home from Paris brings an opera hat called the "Capote." Shirred pink silk front, as big as a chaise top, a crown of double lace (black over white) at the back, a deep cape of the silk, and oh! the flowers. That hat would furnish a westeria for the front porch, hollyhocks for the back yard, bouquets for the parlor mantels, boutonieres for the boys' coats, and then leave some to dry and keep in the Herbarium.

### POKE REDIVIVUS.

"History repeats itself" in no case more surely than in fashion. Hang on to the most obsolete garment you have, the revolving wheel will speedily turn up the emergency that will require it.

Here this March of the centennial year my mind goes back with sorrow to four lovely "sky scraper" bonnets, that years ago went from the garret into the rag bags, and from them, no one knows where.

And here to-day if I had them they would be in the highest sort of fashion.

Let us see—it must be twelve years ago, that the Farewell, vain world! style of hat came in. We had been wearing modest little bonnets placed far back on our heads, in the way the Halo hat is worn now. And severe were the strictures those marvelously worn bonnets received.

A spirit of revenge stirred in the female breast. Dark and dire was the conspiracy against man's comfort, that instigated the invention of the Poke.

I well remember the first one I ever saw. It was the night of a new piece at what was then Laura Keene's, now the Olympic.

A very crowded and fashionable house was in attendance when just before the curtain rose a laugh ran round the auditorium, and looking out of the box I saw a wonderful procession coming down the center aisle. Three ladies, at that time among the most fashionable dressers of their day, were stalking along, crowned by three such hats as we had never seen before. The first comer was Mrs. Chas. Ransom, who was extremely tall, the lady behind was Wm. Wheatley's wife, and she was quite short, and the third was Jerry Bryant's wife, an extraordinary pretty woman.

That brave trio, had got word from Paris of the introduction of the *Poke*, and on this festive occasion they sprung them on an astonished world.

They soared heavenward in front and they dipped earthward behind, they had capes that reached to the small of the back; and whatever outside garment was worn was of little consequence, the capes of those hats covered one like the mantle of charity. Away back inside, where the sufferer's face lived, was placed a bar of flowers. The silken portico stretched a quarter of a yard beyond this—a large bag of silk or lace formed the crown and it was no fool of a bonnet when it went abroad.

Six weeks after I saw the first one, every woman in New York wore a Poke and the universal race of man burst forth in a howl against them.

They obstructed the highways of vision at places of amusement. They were ungraceful, awful, and abominable. No style was ever so anathemized, but they survived, and if anything grew more outrageous in their proportions. With the summer the bag crown of silk was superseded by one of lace.

One of these came to a prompt and ignominous end in the vestibule of Niblo's Garden. It was worn by a well known actress, who enjoyed the regard of a well known actor. The pair went in company with the poke bonnet to a matinee of the "Bronze Horse."

The actor among other possessions had a long lean and active wife, who by some fatality took the children this same day to see the "Bronze Horse." Upon her startled vision then broke the spectacle of the above mentioned pair sailing to seats in the orchestra.

The wonderful hat of the period did not protect the lady's identity. The uglier female perched above detected the well known features in the recesses of the violet shirred silk front which depended the lace bag crown, and that light and pleasing creation, a capote.

With each moment of the performance the injured wife's rage increased, so that at its close she could not very well have done otherwise than to rush for that bonnet as she did.

The collision took place in the vestibule, and at one fell swoop the long wife plucked the entire crown from that hat, and with a dexterous cut knocked all the front part over the astonished actress's face.

There was one moment of indecision. The violet parasol that matched the bonnet described a circle in the air and knocked off a lot of men's hats, ere it landed on the nose of the irate Mrs. F.

Then the husband recovered his senses, had Miss Fan inside a coach and was off with her up Prince street, while the wife informed the curious crowd, that "was her husband, just driving away, and she had taught him not to parade himself in public with that creature again."

The days for matinees attended by ladies in the company of other women's husbands, are by no means over, and as the poke hat with the bag crown is once more among us, this little episode is as likely to be repeated as not.

As a warning, let the fate of the trio above go forth: the wife is dead—the actress is a wreck and the actor is in an insane asylum.

### THE GLORY OF DAGUERREOTYPES.

The Poke Capote is the grand agitation among women just now. It will necessitate a change in the arrangement of hair, and next to the coming eruption of Vesuvius is the greatest convulsion of nature before us for consideration.

However, pokes or pork pies, what does it matter? Only a little while will the fashions vex us, for life's too short for a woman. If you want to see that question illustrated, take up as I did to-day a little book of celebrities, called "Types of the Golden Age," and look on two pictures, one of Sara Coleridge, as a young girl, and one of Sara "some years later."

The fresh graceful girl, whose youthful eyes look out over her rounded cheeks expectantly and brightly, in the one picture, is hidden away in the other out of all sight.

Hollow-chested, wan-cheeked, sunken-eyed and faded, the dancing hair, smoothed down by the theesing hand of time. A close mourning cap, telling what sorrows the years had brought. This was all that was left of *that* after a few brief seasons.

Its dreadful, positively dreadful, and I'm glad the pictures I had taken forty or fifty years ago when in my prime are vanished off the little plates, with nothing to point out where once I sat but a dab of pink paint indicating the cheek I had even then, and a streak of gilt on my fore finger where once was a ring.

That's the advantage of living before the age of photography. Those daguerreotypes had this one merit, they never stayed on the plate long enough to harrow your soul with remorseful comparison.

In their pristine vigor you had to get in a certain position, and catch a flying shifting sight of them.

Then if you closed the case to rest your eyes, away went the mercury with which the plates were prepared, and took you with it.

I've got a lot of these vacant cases containing my relations. Some of them are dead and others alive, but for years I have not known which of them to cry over. As it's just as likely I should be howling over Uncle James (who is alive), thinking its Aunt Miranda (who is not), I don't weep at all ! Therefore, again I say it's a good thing to have lived before photography. •

